# Desmosomal-Type Acantholysis—A New Histologic Pattern Related to Mutations of Genes for Desmosomal Proteins

**DOI:** 10.3390/dermatopathology13020017

**Published:** 2026-04-03

**Authors:** Dieter Metze, Kira Süßmuth, Clemens Metze, Vinzenz Oji, Heiko Traupe

**Affiliations:** 1 International Training Centre for Dermatopathology of the ICDP/UEMS, Dermatopathology Unit, Department of Dermatology, University Hospital Münster, 48149 Münster, Germany; 2 Department of Dermatology, University Hospital Muenster, Von-Esmarch-Str. 56, 48149 Münster, Germany; vinzenz.oji@t-online.de (V.O.);; 3 Helios Klinikum Berlin-Buch, Schwanebecker Chaussee 50, 13125 Berlin, Germany; 4 Department of Cardiology, University Hospital Cologne, Kerpener Straße 62, 50937 Köln, Germany

**Keywords:** desmosomal-type acantholysis, dermatohistologic pattern, desmosomal proteins, genodermatoses, incidental finding

## Abstract

Desmosomes are specialized cell–cell junctions that play a crucial role in maintaining the structural integrity of both cornifying and non-cornifying epithelium. Disruption of desmosomal cohesion in autoimmune, infectious, and other diseases is typically associated with acantholysis, often leading to intraepidermal blisters and erosions. In recent decades, genetic mutations have been identified that impair desmosomal integrity to varying degrees, giving rise to a spectrum of genodermatoses. These conditions, which include palmoplantar keratoderma, epidermolysis bullosa, and ichthyoses, can range from mild to severe, with some forms being syndromic and life-threatening. We investigated dermatopathologic changes in patients with mutations in genes encoding desmosomal proteins seen in consultations at our genodermatoses unit. A series of cases, including keratosis palmoplantaris areata et striata (striated palmoplantar keratoderma type 1), Carvajal–Huerta syndrome, severe dermatitis–multiple allergies–metabolic wasting (SAM) syndrome, ectodermal dysplasia–skin fragility syndrome, and inflammatory peeling skin disease, was examined histologically and, when necessary, immunohistochemically. Findings from our cohort were compared with histopathological consultation cases from our dermatopathology laboratory and previously published cases in the literature. Through these observations, we defined a distinct form of acantholysis associated with desmosomal protein mutations, which we term “desmosomal-type acantholysis.” We outline the spectrum of this newly characterized pattern and highlight its differences from conventional forms of acantholysis. Furthermore, for the first time, we describe incidental cases where “desmosomal-type acantholysis” appears sporadically in solitary acanthoma and in association with a melanocytic nevus.

## Simple Summary

Desmosomes are essential intercellular junctions that ensure epidermal cohesion. While loss of desmosomal adhesion is mainly associated with autoimmune and infectious blistering disorders, the histopathologic consequences of inherited defects in desmosomal proteins are less clearly defined. In this study, we systematically analyzed skin biopsies from patients with genetically confirmed desmosomal disorders, including palmoplantar keratoderma, cardiocutaneous syndromes, skin fragility syndromes, and inflammatory cornification disorders. We identified a reproducible and distinctive pattern of keratinocyte dissociation that differs from conventional pemphigus-type acantholysis. We designate this pattern as “desmosomal-type acantholysis” and delineate its morphologic spectrum and diagnostic features. In addition, we report incidental occurrences of this pattern in solitary acanthoma and in association with melanocytic nevi. Recognition of desmosomal-type acantholysis provides a useful histopathologic clue to underlying desmosomal gene defects and has diagnostic relevance for dermatopathology practice.

## 1. Introduction

Genodermatoses with epidermal differentiation disorders represent a highly heterogeneous group of diseases that are often difficult to diagnose both clinically and histologically [1]. Given that many of these conditions have a syndromic presentation and can be associated with potentially life-threatening complications, a prompt diagnosis is crucial, which includes targeted molecular testing.

In recent years, our team of the Network for Ichthyoses and Related Keratinization Disorders (NIRK), Reference Center for Ichthyoses and Palmoplantar Keratoderma (ReCIP), and ERN Skin Reference Centre Münster has systematically analyzed histological patterns and diagnostic criteria in a large cohort of well-characterized keratinization disorders [2]. This work has also included the investigation of diseases linked to mutations in genes encoding desmosomal proteins.

Desmosomes are specialized cell–cell junctions that provide strong adhesion and flexibility between adjacent epithelia and myocardial cells. In the skin and mucosa, they play a critical role in maintaining the structural integrity and cohesion of the cornifying and non-cornifying epithelium [3].

Desmosomes are dynamic adhesion complexes that function by anchoring intermediate filaments to the plasma membrane, supporting epidermal strength. Desmosomes are composed of transmembrane adhesion proteins of the cadherin family, i.e., desmogleins (DSGs1–4) and desmocollins (DSC 1–3), and intracellular anchor proteins, such as plakoglobins (PKP1–3), plakophilins, desmoplakin (DSP), and corneodesmosin, which connect the desmosomes to the keratin cytoskeleton [3].

Desmosomes also play a critical role in heart function, as some mutations in genes encoding desmosomal proteins are associated with non-syndromic cardiomyopathy and cardiocutaneous syndromes. These findings highlight the dual importance of desmosomes in maintaining both skin and cardiac integrity (Table 1) [4,5,6].

Desmosomal proteins serve functions beyond merely maintaining cell–cell adhesion. They also regulate epidermal differentiation, proliferation, and homeostasis. By interacting with key molecules in the Wnt/β-Catenin, TGF-β/SMAD, Hippo, NF-κB, and EGFR signalling pathways, they influence critical cellular processes. Mutations or dysregulation of these proteins can disrupt these pathways, contributing to inflammatory skin diseases, blistering disorders, and cancer development. Accordingly, certain genodermatoses with a strong inflammatory component, such as severe dermatitis–multiple allergies–metabolic wasting (SAM) syndrome, can be effectively targeted with anti-inflammatory biologic therapies [7,8,9,10].

Disruption of the desmosomal cohesion usually leads to intraepidermal blisters and erosions. Histologically, a separation of keratinocytes from each other can be seen, which is described as acantholysis (Greek, “loosening of spines”). Acantholytic keratinocytes become compact and hypereosinophilic with a dense nucleus. This classic type occurs in the stratum spinosum, where polygonally shaped keratinocytes are affected. In contrast, the detachment of flat keratinocytes below the stratum corneum in the stratum granulosum results in the separation of elongated keratinocytes below the stratum corneum [11].

Acantholysis occurs in autoimmune diseases of the pemphigus group, where autoantibodies against desmosomal proteins disrupt their function. In Pemphigus vulgaris, autoantibodies against desmoglein 3 can be detected, while in Pemphigus foliaceus, autoantibodies target Desmoglein 1, which is predominantly expressed in the upper layers of the epidermis. In paraneoplastic pemphigus, a cocktail of antibodies attacks both transmembrane adhesion proteins, such as desmogleins and desmocollins, and intracellular anchor proteins, among them desmoplakin and others [12].

Acantholysis can also be caused by bacterial exfoliative toxins that cleave Desmoglein 1 in the upper epidermis, mimicking pemphigus foliaceus. Examples are superficial skin infection as seen in impetigo and systemic dissemination of exfoliative toxins in Staphylococcal Scalded Skin Syndrome (SSSS). Histologic distinction from superficial forms of pemphigus (pemphigus foliaceus, IgA pemphigus) is not possible; thus, microbiological analysis, toxin detection and immunofluorescence are crucial for the differential diagnosis [11].

In addition, many other diseases exhibit histologic features of acantholysis, even if they do not always present with blistering or crusting. Examples include genodermatoses such as Darier disease, Hailey–Hailey disease, Galli–Galli disease, and certain forms of epidermolysis bullosa with epidermal manifestations. Acantholysis may also be observed in Grover’s disease, pityriasis rubra pilaris, and as an artificial consequence of physical trauma or chemical damage (e.g., Cantharidin, EMLA^®^). Moreover, neoplasms such as solar keratosis and squamous cell carcinoma frequently exhibit acantholysis [13].

Focal formation of acantholytic epithelium, with or without dyskeratosis, may also occur as an incidental finding in normal skin, epidermal nevi, benign acanthomas, and, incidentally, in benign and malignant neoplasms, as well as unrelated inflammatory skin diseases. These changes can appear either within the primary lesion or in the surrounding epithelium [14,15,16].

In recent decades, genetic mutations in desmosomal proteins have been identified that disrupt desmosomal coherence to varying degrees and cause a variety of genodermatoses categorized as palmoplantar keratoderma, epidermolysis bullosa, and ichthyoses, some of which are syndromic and life-threatening (Table 1).

This article presents the dermatopathologic changes observed in patients with mutations in genes encoding desmosomal proteins from consultations in our genodermatoses unit. Through these observations, we define a distinct form of acantholysis, which we refer to as “desmosomal-type acantholysis”. The spectrum of this newly characterized pattern is demonstrated, and its differences from conventional forms of acantholysis are discussed. Beyond that, for the first time, we describe incidental cases where “desmosomal-type acantholysis” occurs randomly in association with other dermatoses, as well as spontaneously arising in benign acanthoma.

## 2. Patients and Methods

A series of patients with mutations in genes encoding desmosomal proteins from consultations at our genodermatoses unit were histologically examined using H&E-stained routine paraffin sections. When necessary, additional immunohistochemical analyses were performed, including Desmoglein 1 and Keratin 16 staining (Desmoglein 1: SantaCruz, monoclonal, 18D4; Cytokeratin 16: Zytomed/Diagnostic Biosystems, monoclonal, LL025). The cases involved clinically and genetically well-characterized diagnoses, including keratosis palmoplantaris areata et striata (striated palmoplantar keratoderma type 1), Carvajal–Huerta syndrome, severe dermatitis–multiple allergies–metabolic wasting (SAM) syndrome, and inflammatory peeling skin disease. The case of ectodermal dysplasia–skin fragility syndrome was analyzed using an Epon-embedded semithin section of the first published case, kindly provided by J. McGrath [17]. As an incidental finding, one case of solitary acanthoma and melanocytic nevus was identified. The findings from our cohort were compared with histopathological consultation cases from our dermatopathology laboratory and previously published cases in the literature.

## 3. Results

### 3.1. Palmoplantar Keratoderma with Desmosomal-Type Acantholysis

#### 3.1.1. Striate Palmoplantar Keratodermas, Type 1 (SPPK1)

SPPK, type 1, also known as keratosis palmoplantaris areata et striata (syn.: Brünauer–Fuchs–Siemens syndrome), is an autosomal dominant disorder characterized by linear keratotic bands on the palms and fingers and island-like areas of hyperkeratosis on the soles over pressure points. Lesions evolve in adolescence or early adulthood and are exacerbated by mechanical stress. In contrast to other forms of striate palmoplantar keratodermas, there are no systemic symptoms [1].

The underlying genetic defect in Keratosis palmoplantaris areata et striata, type 1 is a mutation of Desmoglein 1 (Dsg1). Ultrastructurally, desmosomes appear diminished and rudimentary, keratin filaments are aggregated, and keratohyalin granules are enlarged and malformed [18], while perinuclear aggregation of keratin filaments seems more marked in desmoplakin-associated SPPK, type 2 [19].

Importantly, DSG1 mutations in DSG1 are associated not only with striate hyperkeratosis but also with diffuse palmoplantar keratoderma (PPK); however, definitive genotype–phenotype correlations remain to be established [20]. Of further interest, carriers of heterozygous mutations in *DSG1* display PPK only, while the offspring of two such heterozygous carriers can be affected by a life-threatening condition known as severe dermatitis–multiple allergies–metabolic wasting syndrome (SAM syndrome) [21].

**Figure 1:**
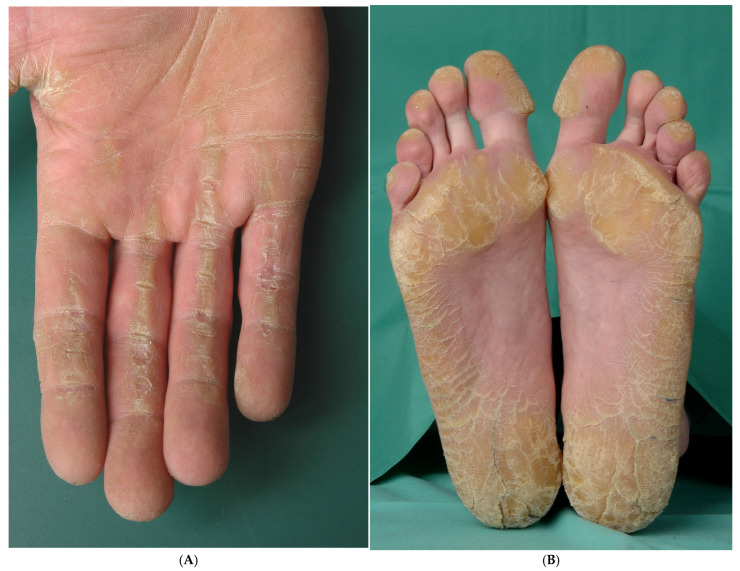

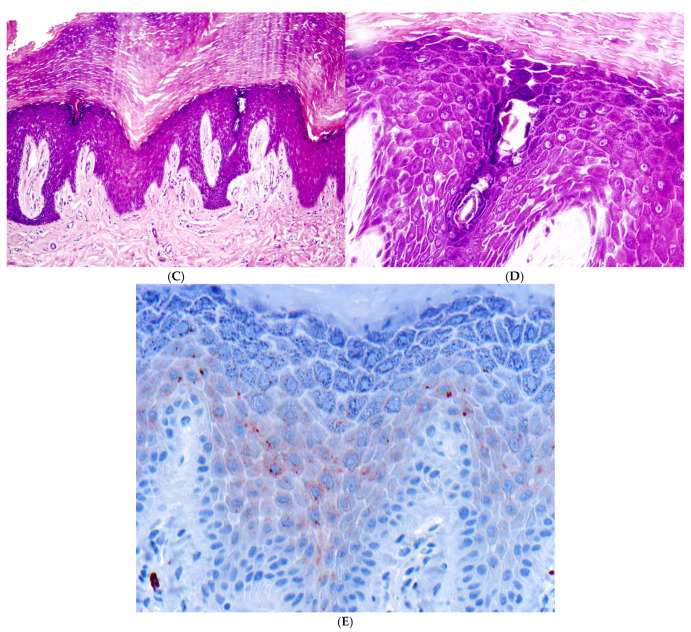
Striate palmoplantar keratodermas, type 1 (SPPK1). (**A**,**B**) A 36-year-old man, family history-positive, with no associated symptoms. Since childhood, palmoplantar keratoses have presented in a striate pattern on the hands and an areata-like pattern on the soles of the feet over the pressure points. (**C**) There is acanthosis and orthohyperkeratosis of the epidermis, papillomatosis, and almost no inflammatory infiltrate (H&E, original magnification: 100×). (**D**) Widening of the intercellular spaces in the stratum spinosum and stratum granulosum. The keratinocytes partially lose their intercellular connections. The cell shape remains polygonal in the stratum spinosum and flattened in the stratum granulosum (H&E, original magnification: 200×). (**E**) Immunostaining for Desmoglein 1 demonstrates a loss of the membranous staining pattern, with only a discrete residual granular immunoreactivity in the cytoplasm. (Immunostaining for Desmoglein 1, original magnification: 200×).

Histopathology: There is acanthosis and papillomatosis, as well as orthohyperkeratosis and hypergranulosis. In the stratum spinosum and stratum granulosum, a widening of the intercellular spaces is observed. The keratinocytes partially lose their intercellular connections. There is no detachment of keratinocytes. The cell shape remains polygonal in the stratum spinosum and flattened in the stratum granulosum, without rounding. Since these changes are discrete and focal, with variable expression, step sections can be recommended. There is no paleness of the keratinocytes, no serum exudation, no parakeratosis, and almost no dermal inflammatory infiltrate (Figure 1).

Immunohistochemistry with antibodies against Desmoglein 1 shows a complete loss of the typical membranous staining pattern, while some granular residuals are left in the cytoplasm (Figure 1E).

#### 3.1.2. Palmoplantar Keratoderma with Left Ventricular Cardiomyopathy and Woolly Hair (Carvajal Syndrome)

Carvajal syndrome is characterized by palmoplantar keratoderma (PPK) of the areata and striated type, along with woolly hair and cardiomyopathy. This rare disorder arises from mutations in the desmoplakin gene, which is inherited in an autosomal recessive manner. Desmoplakin plays a crucial role in desmosomes and interacts with intermediate filaments in the heart, hair, and skin [22,23].

**Figure 2:**
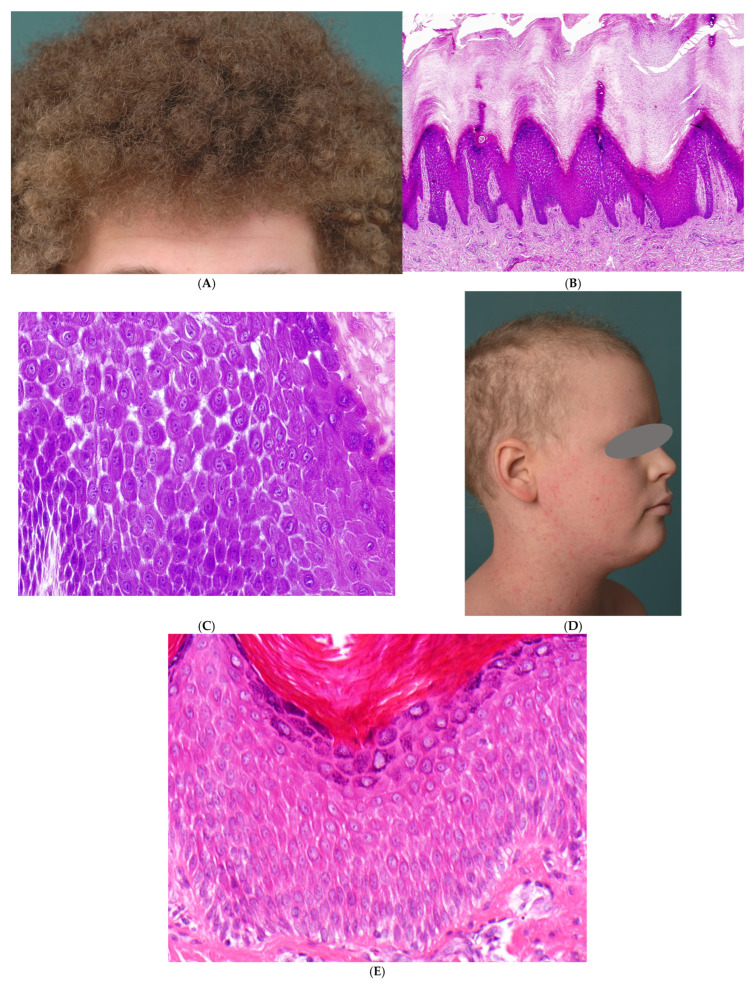
Carvajal syndrome. (**A**) A 16-year-old male patient. The diagnosis was genetically confirmed by J. Fischer, Medical Director of the Institute of Human Genetics at the University Medical Center Freiburg, when the patient was 13 years old. (**B**) Acanthosis with undulating surface, orthohyperkeratosis, papillomatosis, discrete inflammatory infiltrate (H&E, original magnification: 100×). (**C**) Incomplete separation of the keratinocytes with hypereosinophilic cytoplasm, reduction in intercellular bridges and partial loss of polygonal shape (H&E, original magnification: 400×). (**D**) A 6-year-old boy with hairs that are short and thinned. An implantable cardioverter-defibrillator was required, followed by a heart transplantation. (**E**) The keratinocytes show minimal separation and a reduction in intercellular bridges, accompanied by a hypereosinophilic cytoplasm (H&E, original magnification: 400×).

In Carvajal syndrome, PPK typically does not manifest until infancy, may be very mild, and is not always striated; it can also extend over the Achilles tendon. Scaly hyperkeratoses may occasionally develop on other areas of the body, such as the knees and elbows. While woolly hair is generally a prominent symptom at birth (Figure 2A), other hair abnormalities, including pili torti and short, thin or dry hairs (Figure 2D), can also occur [1].

The onset of cardiac manifestations can occur anywhere from early childhood to adulthood. It usually results in the enlargement of the left and/or right ventricle, leading to heart failure and ventricular arrhythmias, which significantly increase the risk of sudden cardiac death [8].

Genetic defects in desmoplakin have been associated with a diverse array of phenotypes. Heterozygous carriers may exhibit isolated arrhythmogenic right ventricular cardiomyopathy without any cutaneous manifestations. Vice versa, compound heterozygote patients with one null allele and one missense mutation result in skin fragility and alopecia in the absence of cardiac anomalies. A complete loss of the tail domain in desmoplakin results in acantholytic epidermolysis bullosa [24].

Clinical presentation of Carvajal syndrome is very heterogeneous and shows overlaps with Naxos syndrome, which is also a syndromic PPK characterized by woolly hair and congestive heart failure due to mutations in plakoglobin [5].

##### Histopathology

There is orthohyperkeratosis and hypergranulosis, accompanied by acanthosis and papillomatosis. The keratinocytes exhibit incomplete separation, along with a hypereosinophilic cytoplasm, a reduction in intercellular bridges, and a partial loss of their polygonal shape, though without complete rounding. A discrete inflammatory infiltrate is present in the dermis (Figure 2B,C,E).

### 3.2. Ichthyoses with Desmosomal-Type Acantholysis

#### 3.2.1. Severe Dermatitis–Multiple Allergies–Metabolic Wasting Syndrome (SAM Syndrome)

SAM syndrome was identified in 2013 by Liat Samuelov et al. as a severe, life-threatening genodermatosis [25]. The acronym stands for severe dermatitis–multiple allergies–metabolic wasting syndrome. It is caused by mutations of Desmoglein 1 (DSG1). The disease is inherited in an autosomal recessive manner; heterozygous carriers of the DSG1 mutation only develop striate palmoplantar keratoderma [21]. Later, a desmoplakin mutation with autosomal dominant inheritance was identified [26].

**Figure 3:**
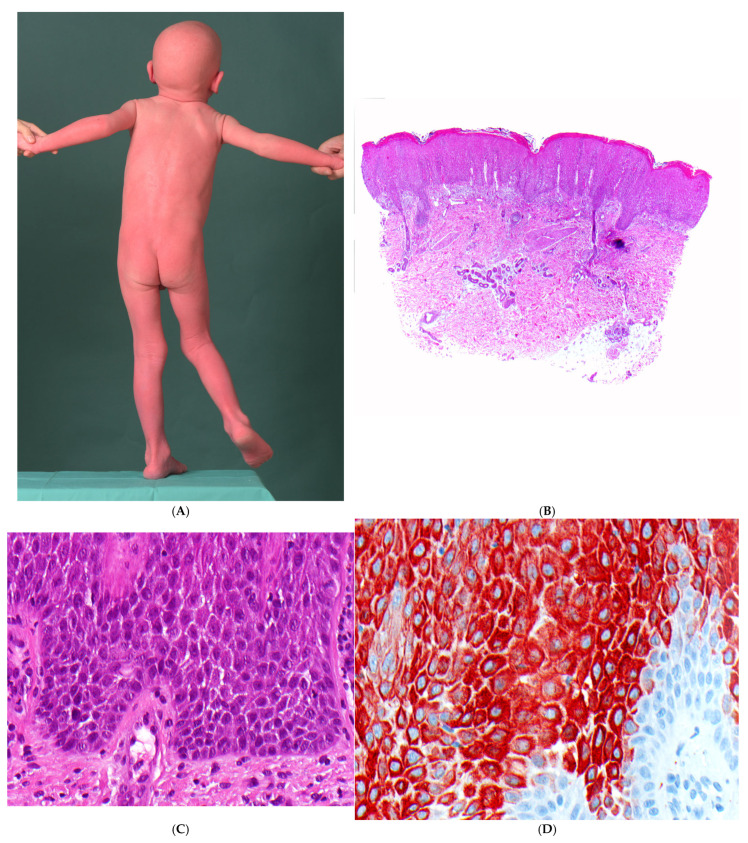
Severe dermatitis–multiple allergies–metabolic wasting syndrome (SAM syndrome). (**A**) A 5-year-old boy with a highly pruritic erythroderma, atrichia and palmoplantar keratoderma. A dominant missense mutation in the gene for desmoplakin has been identified by J. Fischer, Medical Director of the Institute of Human Genetics at the University Medical Center Freiburg. (**B**) Psoriasiform dermatitis mimicking psoriasis vulgaris (H&E, original magnification: 40×). (**C**) At higher magnification, keratinocyte dehiscence and a reduction in intercellular bridges can be observed (H&E, original magnification: 200×). (**D**) Keratin 16, a marker of hyperproliferative keratinocytes, is strongly expressed and highlights the separation of keratinocytes (Immunostaining for keratin 16, original magnification: 200×).

Clinically, newborns present with ichthyosiform erythroderma, similar to cases with autosomal recessive congenital ichthyoses (ARCI), Netherton syndrome or peeling skin disease (Figure 3A). Other symptoms include pruritus, hypotrichosis, food allergies with increased IgE, dysphagia, reduced growth and recurrent skin and respiratory tract infections. Pustule formation, palmoplantar keratoses, onychodystrophy, dental anomalies, cardiac anomalies and eosinophilic esophagitis occur to varying degrees. There is marked inter- and intrafamilial variability [27]. The accompanying inflammation can be explained by pro-inflammatory activity in keratinocytes in connection with impaired barrier function and downregulated blockade of signal transduction pathways [28]. In addition, the intracytoplasmic portion of Dsg1 blocks the RAS-RAF signalling pathway and thus influences epidermal differentiation [29].

##### Histopathology

Histologically, a superficial lymphocytic dermatitis with hyperplastic epidermis, parakeratosis and neutrophilic granulocytes is seen, which strongly resembles psoriasis (psoriasiform dermatitis). However, dilated intercellular spaces of the epidermis without blistering are typical (Figure 3B,C), and a reduction in intercellular bridges in the stratum spinosum and stratum granulosum can be demonstrated by keratin immunostaining (Figure 3D). In cases involving desmoglein mutations, the cell membranes of keratinocytes show no immunoreactivity for DSG1, with only a fine granular cytoplasmic signal remaining. At the ultrastructural level, mature desmosomes are absent in the acantholytic areas of the stratum spinosum and stratum granulosum, replaced instead by half-split desmosomes [25].

### 3.3. Skin Fragility Syndrome with Desmosomal-Type Acantholysis

#### 3.3.1. Ectodermal Dysplasia–Skin Fragility Syndrome (McGrath Syndrome)

Ectodermal dysplasia–skin fragility syndrome, or McGrath syndrome, is an autosomal recessive condition classified under epidermolysis bullosa. Newborns typically display peeling, reddish skin and blisters on their soles. As patients grow, they experience diffuse palmoplantar hyperkeratosis, sometimes verruciform with painful cracks, skin erosions, particularly around the mouth, and other ectodermal abnormalities such as growth delay, reduced sweating, hypotrichosis or alopecia, and nail dystrophy. Notably, unlike some desmosomal disorders, there is no cardiac involvement [17]. Further observations in some individuals included pruritus, failure to thrive with low height/weight centiles, follicular hyperkeratosis, walking difficulties, dysplastic dentition and recurrent chest infections [30].

Mutations in the plakophilin-1 gene result in the loss of plakophilin 1 (PKP1), crucial for desmosomal protein recruitment and keratin interaction [31].

##### Histopathology

**Figure 4:**
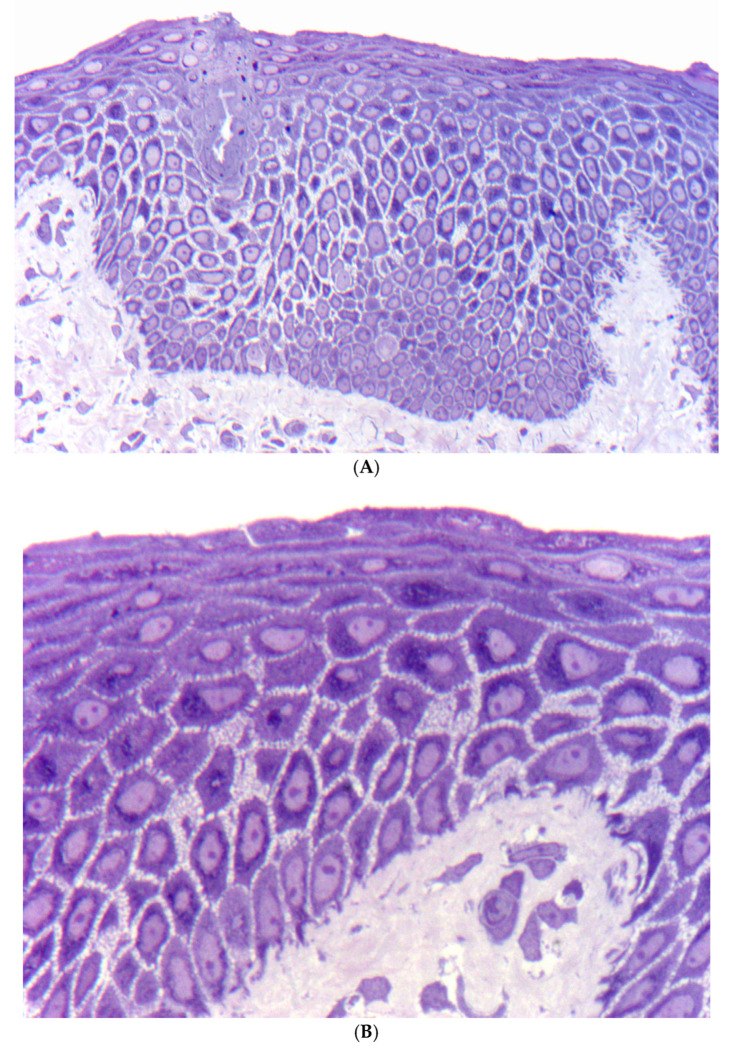
Ectodermal dysplasia–skin fragility syndrome. Original slide from a 6-year-old boy (Courtesy of John McGrath) [17]. (**A**) Semithin section of an Epon-embedded skin biopsy reveals epidermal thickening and widened intercellular spaces in the suprabasal layers (Toluidine blue, original magnification: 200×). (**B**) At higher magnification, intracytoplasmic globules representing paranuclear clumping of the keratin skeleton are evident (Toluidine blue, original magnification: 400×).

Light microscopy reveals epidermal thickening and widening of keratinocyte intercellular spaces, causing clefting and blister formation [32]. Of note is a circumscribed hypereosinophilic keratin condensation in the paranuclear area of suprabasal keratinocytes (Figure 4). A complete lack of plakophilin-1 immunostaining is highly diagnostic [33]. Electron microscopy indicates a loss of keratinocyte adhesion, with reduced and poorly developed desmosomes, especially in the lower suprabasal layers. The inner and outer desmosomal plaques are poorly formed. Affected keratinocytes exhibit detachment of keratin filaments from desmosomes and perinuclear condensation [34].

#### 3.3.2. Peeling Skin Syndrome (PSS)

Peeling Skin Syndrome (PSS) is characterized by spontaneous peeling of the stratum corneum without bleeding or pain [8,35]. PSS includes several subtypes caused by mutations in different genes, e.g., the TGM5 gene that primarily affects the skin on palms and soles. Generalized PSS occurs in a non-inflammatory type, designated type A (familial continual skin peeling, keratolysis exfoliativa congenitale) and is caused by a mutation in *CHST8* [36].

The inflammatory type B, designated as PSS B (inflammatory peeling skin syndrome), is due to mutations of corneodesmosin [37]. Corneodesmosin is an important adhesion protein that is expressed in the extracellular sections of the desmosomes in the stratum corneum of the epidermis and in the inner hair root sheath of the hair follicles. Accordingly, autosomal dominant mutations in other domains of corneodesmosin cause hypotrichosis simplex.

**Figure 5:**
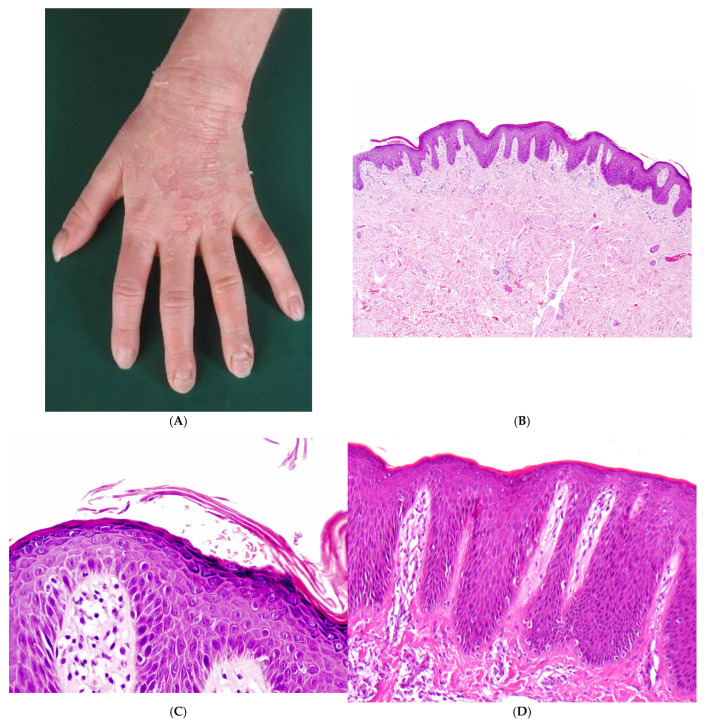
Peeling skin syndrome, inflammatory type (type B). (**A**) A 4-year-old girl with isolated erythematous lesions with flaccid peeling and episodic detachment of the nail plates (onychomadesis). (**B**) Epidermal hyperplasia with prominent rete ridges, accompanied by a superficial and perivascular lymphocytic infiltrate (H&E, original magnification: 40×). (**C**) Mild hyperkeratosis with focal parakeratosis, along with detachment of the stratum corneum and scattered flat acantholytic corneocytes (H&E, original magnification: 200×). (**D**) In another biopsy, a pronounced psoriasiform dermatitis, characterized by an elongated papillary dermis with dilated vessels and a thin, compact stratum corneum, without signs of acantholysis (H&E, original magnification: 100×).

PSS type B manifests at birth with ichthyosiform erythroderma and is characterized by lifelong patchy peeling of the entire skin, accompanied by severe pruritus. Isolated erythematous lesions exhibit flaccid peeling, resulting in superficially denuded, burning red patches with a characteristic peripheral collarette (Figure 5A). It persists throughout life with seasonal fluctuations. In addition, episodic detachment of the nail plates (onychomadesis) may occur. The hair status is unremarkable, apart from a temporary slight epilation of the fine hair.

The simultaneous disruption of the barrier leads to inflammation with severe itching, urticaria, angioedema, food allergies and asthma with increased IgE levels and eosinophilia in the blood.

##### Histopathology

The epidermis is hyperplastic with prominent rete ridges. There is mild hyperkeratosis with focal parakeratosis and thinned stratum granulosum (Figure 5B). Some biopsies show focal detachment of the stratum corneum, and in some cases, the stratum corneum is completely absent (Figure 5C). However, these changes are not always visible on a paraffin section. There are superficial and perivascular lymphocytic infiltrates with individual neutrophils, which are also found in the stratum corneum. Other cases exhibit a psoriasiform and spongiotic dermatitis or a pronounced psoriasiform dermatitis with variably dilated vessels (Figure 5D). Immunostaining for corneodesmosin and LEKTI may help to distinguish between Netherton syndrome and PSS type B [37].

Electron microscopy shows that a loss of corneodesmosomes can be demonstrated, which is associated with intercellular splitting of the corneocytes from the stratum granulosum [37].

## 4. Desmosomal-Type Acantholysis in Solitary Acanthoma and as an Incidental Finding

### 4.1. Solitary Acanthoma

Acanthomas present as well-demarcated, keratotic papules or plaques, often resembling verruca vulgaris or seborrheic keratosis clinically. Histologically, they manifest as flat, acanthotic, and hyperkeratotic epidermal tumours, characterized by specific cytological features such as clear cells (clear cell acanthoma), enlarged keratinocytes (large cell acanthoma), epidermolytic keratinocytes (epidermolytic acanthoma), and dyskeratotic keratinocytes (dyskeratotic acanthoma) [16].

**Figure 6:**
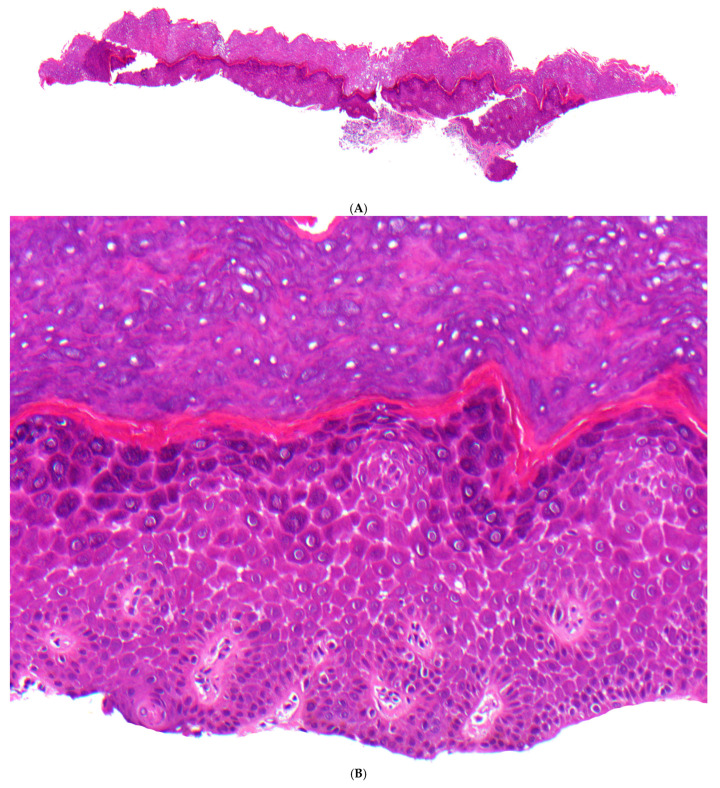

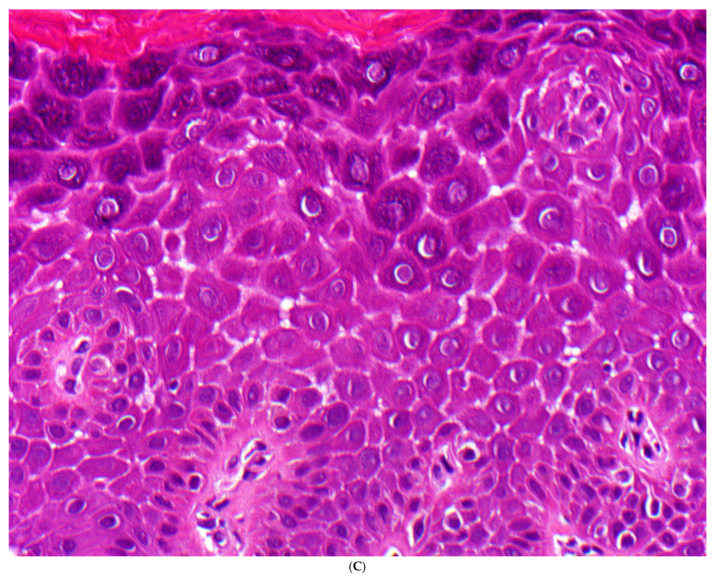
Incidental finding of desmosomal-type acantholysis in solitary acanthoma. A 65-year-old man with clinical suspicion of verruca vulgaris, shave biopsy from the back of the hand. (**A**) Acanthotic epidermis with a prominent stratum granulosum and massive orthohyperkeratosis (H&E, original magnification: 20×). (**B**) Focally widened intercellular spaces in the stratum spinosum and lower stratum granulosum (H&E, original magnification: 200×). (**C**) Reduction in intercellular bridges of the keratinocytes and hypereosinophilic cytoplasm (H&E, original magnification: 400×).

Notably, we have observed solitary benign acanthomas with focal widening of intercellular spaces in the stratum spinosum and lower stratum granulosum. The intercellular bridges between keratinocytes appeared reduced, while the cytoplasm exhibited pronounced hypereosinophilia. Otherwise, the stratum granulosum was prominent, and the horny layer displayed marked orthohyperkeratosis, with no evidence of dyskeratosis or parakeratosis (Figure 6).

### 4.2. Incidental Finding Randomly Associated with a Melanocytic Nevus

Various histologic patterns, such as epidermolytic hypergranulosis or focal acantholytic dyskeratosis, can occur incidentally and focally as non-specific findings in the epidermis overlying or adjacent to an unrelated lesion. These changes are common and have been reported in conditions such as melanocytic nevi, seborrheic keratoses, actinic keratosis, squamous cell carcinoma, and melanoma [16].

**Figure 7:**
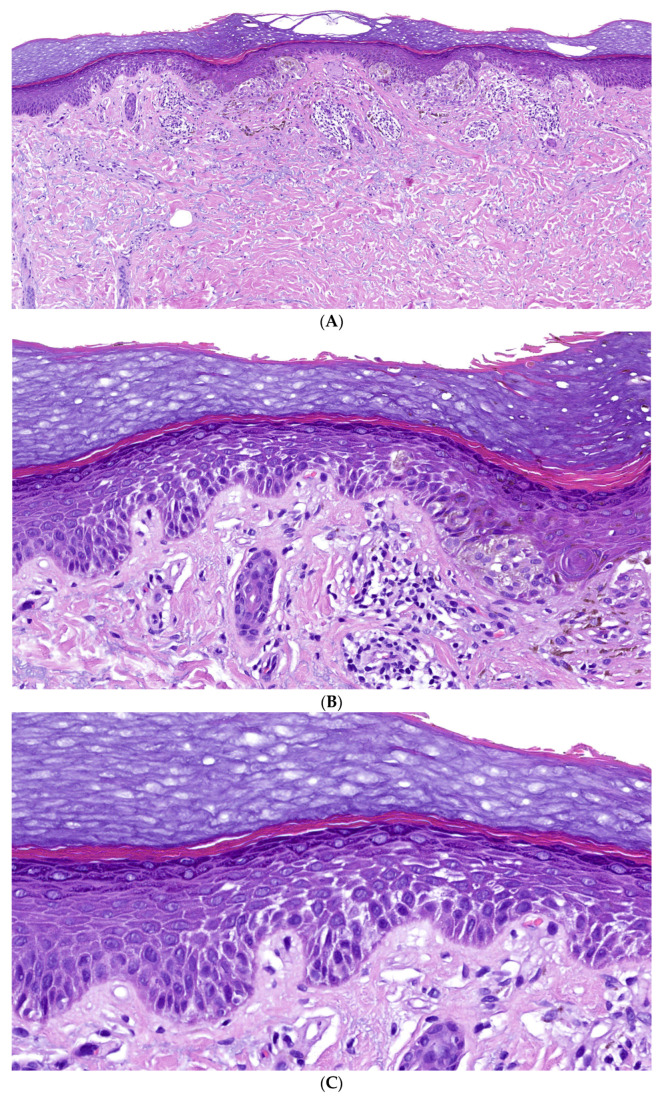
Incidental finding of desmosomal-type acantholysis in a melanocytic nevus. A 67-year-old woman with clinical diagnosis of melanocytic nevus, excision biopsy from the wrist. (**A**) Melanocytic nevus of the junctional type on acral site (H&E, original magnification: 40×). (**B**) Focal separation of suprabasal keratinocytes with cleft formation on one side of the lesion (H&E, original magnification: 100×). (**C**) Keratinocytes appear eosinophilic with reduced intercellular bridges. Well-developed stratum granulosum and orthokeratototic horny layer (H&E, original magnification: 200×).

In such incidental cases, the alterations are typically confined to the epidermis overlying only one or two dermal papillae. A similar phenomenon, characterized by suprabasal keratinocyte separation with cleft formation and reduced intercellular bridges, was observed by us on one side of a junctional melanocytic nevus (Figure 7).

## 5. Discussion

### 5.1. Desmosomal-Type Acantholysis, Definition of Term

In a series of syndromic and non-syndromic genodermatoses associated with mutations in genes encoding desmosomal proteins, we analyzed histological changes in the affected epidermis and compared our findings with those reported in the literature (Table 2) [4]. Through this analysis, we identified a distinct form of acantholysis, which we propose to call desmosomal-type acantholysis (Table 2).

The hallmark of desmosomal-type acantholysis is widening of the intercellular spaces with partial loss of intercellular bridges in the suprabasal epidermis. Although similar changes may also be observed in spongiotic dermatitis, the pattern of desmosomal acantholysis consistently lacks keratinocyte pallor, vesiculation, blister formation, and serous exudation. Despite the dehiscence, keratinocytes retain their polygonal shape in the stratum spinosum and a flattened morphology in the stratum granulosum or horny layer to some extent, with intact nuclei and preserved differentiation. Their cytoplasm remains eosinophilic, occasionally exhibiting hypereosinophilia and/or eosinophilic clumping. Desmosomal-type acantholysis is frequently accompanied by variable degrees of inflammation, acanthosis, papillomatosis, orthohyperkeratosis, and hypergranulosis.

At the ultrastructural level, “desmosomal-type acantholysis” exhibits rudimentary desmosomal structures, disruption of desmosome–keratin interactions, as well as condensation, clumping or perinuclear collapse of the keratin filament network [18,19]. Accordingly, immunofluorescence studies showed altered expression profiles of affected desmosomal proteins [4].

### 5.2. Histologic Spectrum of Desmosomal-Type Acantholysis and Differential Diagnoses

In our histologic study, we observed that desmosomal-type acantholysis is associated with a spectrum of histologic changes, which can be differentiated into four histologic subgroups. (Table 3).

#### 5.2.1. Category 1: Widening of the Intercellular Spaces in the Stratum Spinosum and Occasionally in the Stratum Granulosum

Several keratoderma present with a striate and/or focal pattern (see Table 3), some of them may be associated with a syndromic disease, e.g., Naxos syndrome and Carvajal syndrome (see below). In general, predominantly striate and/or focal PPK are a clinical clue for inherited disease with mutations of desmosomal proteins, histologically featuring desmosomal-type acantholysis.

In striated palmoplantar keratoderma type 1 (SSPK1) and palmoplantar keratoderma associated with Carvajalsyndrome, the stratum spinosum of the epidermis, and occasionally the stratum granulosum, show widened intercellular spaces along with a partial loss of intercellular bridges. Notably, this occurs without vesiculation or blister formation. The keratinocytes remain partially dehiscent yet retain their polygonal shape to some extent, with intact nuclei and preserved differentiation. Their cytoplasm remains eosinophilic, sometimes exhibiting hypereosinophilia. However, the acantholytic changes can be very subtle or even absent in certain areas of the biopsy, making step-sectioning essential for accurate assessment. Additional histopathologic features include acanthosis, papillomatosis, orthohyperkeratosis, and hypergranulosis. In the papillary dermis, a mild lymphocytic infiltrate may be present to a variable extent. Similar histologic findings have been reported in the literature for diffuse non-epidermolytic PPK caused by nonsense mutation of DSG1, SPPK type 2 related to desmoplakin 1 mutations, and Naxos syndrome [18,19,20,38,39].

Similar histologic changes have been described in other genodermatoses with mutations encoding desmosomal proteins. Acantholytic ectodermal dysplasia caused by mutations of JUP encoding Plakoglobin presents with epidermal skin fragility, diffuse PKK, curly hair, nail dystrophy, and no cardiac symptoms at a younger age [40].

Lethal acantholytic epidermolysis bullosa (EBLA) is caused by homozygous or compound heterozygous mutation in the desmoplakin gene (DSP), characterized by lethal generalized epidermolysis, universal alopecia, and anonychia, with or without cardiac involvement [24,41]. Lethal congenital epidermolysis bullosa is related to a complete loss of plakoglobin, where patients suffer from severe congenital skin fragility with generalized epidermolysis and massive transcutaneous fluid loss, but apparently no cardiac dysfunction [42].

Beyond that, histologic differential diagnoses of desmosomal-type acantholysis include other inherited disorders, such as epidermolysis bullosa simplex and Kindler syndrome. However, since these conditions primarily affect basal keratinocytes, they are not considered further in the differential diagnosis [43].

A key differential diagnosis includes non-inherited forms of acantholysis, such as autoimmune blistering disorders. In pemphigus diseases, the epidermis exhibits clefting and blister formation while preserving normal cornification. The keratinocytes are fully acantholytic, appearing rounded with pyknotic nuclei and uniformly intense hypereosinophilic cytoplasm.

Other forms of intraepidermal blistering include infections caused by herpes viruses and other viral pathogens. In these cases, characteristic histologic features such as ballooning (intracellular edema), reticular degeneration, and blistering, along with typical viropathic nuclear changes, facilitate a clear diagnosis.

Spongiotic dermatitis, as seen in eczematous diseases, is also characterized by the widening of intercellular spaces within the epidermis. However, in spongiotic dermatitis, keratinocytes exhibit a pale cytoplasm due to intracellular edema, intraepidermal vesiculation, serum exudation, and crust formation. Additionally, these changes are associated with the loss of the stratum granulosum and prominent parakeratosis. Because many characteristic criteria may be absent in early stages of spongiotic dermatitis, a definitive histological differentiation is not always possible.

Artifacts, such as friction blisters, show blister formation in the superficial epidermis, accompanied by pale or necrotic keratinocytes in the base of the blister.

#### 5.2.2. Category 2: Widening of the Intercellular Spaces with Psoriasiform Dermatitis

Notably, desmosomal-type acantholysis is associated with psoriasiform dermatitis as seen in severe dermatitis–multiple allergies–metabolic wasting (SAM) syndrome. In addition to a superficial lymphocytic dermatitis with epidermal hyperplasia, parakeratosis, and neutrophilic granulocytes, key histopathological findings include dilated intercellular spaces within the epidermis without blistering, as well as keratinocyte dehiscence with a reduction in intercellular bridges.

In general, SAM syndrome exhibits histologic features that closely resemble psoriasis vulgaris or chronic dermatitis within the context of atopic eczema. For histological differentiation, immunohistochemical analysis of keratinocytes in SAM syndrome reveals a lack of immunoreactivity for DSG1 or Desmoplakin along the cell membranes, with only a faint granular cytoplasmic signal remaining.

Another mutation in the Desmoplakin gene leads to erythrokeratodermia-cardiomyopathy syndrome, which also causes a similar psoriasiform dermatitis; however, widening of intercellular spaces has only been observed at the ultrastructural level [44].

Additionally, distinguishing other genodermatoses with a psoriasiform pattern can be challenging (Table 3). Notably, Netherton syndrome, beyond its psoriasiform features, including intraepidermal neutrophils, may also exhibit subcorneal or intracorneal splitting [45]. Since Netherton syndrome is caused by mutations in the SPINK5 gene, which encodes the lympho-epithelial Kazal-type-related inhibitor (LEKTI), the absence of immunostaining for this serine protease inhibitor aids in its differential diagnosis.

Similarly, distinguishing CHILD syndrome and MALT1 deficiency syndrome, two other genetic disorders with a psoriasiform pattern, can be facilitated by immunostaining for desmosomal proteins, keratin 10, LEKTI, adipophilin, and MALT1 [26,46].

#### 5.2.3. Category 3: Widening of Intercellular Spaces with Intracytoplasmic Hypereosinophilic Globules in Suprabasal Keratinocytes

In ectodermal dysplasia–skin fragility syndrome (McGrath syndrome), desmosomal-type acantholysis is characterized by the paranuclear aggregation of the keratin cytoskeleton, leading to the formation of prominent hypereosinophilic globules within suprabasal keratinocytes. In addition, the epidermis reveals epidermal thickening and widening of keratinocyte intercellular spaces, with some clefting. (Figure 5). A complete lack of plakophilin-1 immunostaining is highly diagnostic [33].

Similarly, larger keratin clumping is observed in *Pachyonychia congenita* related to mutations in the genes encoding keratin 6, 16, or 17. In contrast to ectodermal dysplasia–skin fragility syndrome, the eosinophilic globules and the rest of the cytoplasm are paler, and the dehiscence of keratinocytes is lacking. Beyond that, a massive hyperkeratosis and patchy hypergranulosis allow for differentiation.

Similar paranuclear eosinophilic inclusions have been observed in a solitary acanthoma, referred to as hyaline inclusion acanthoma. In this condition, eosinophilic globules are located beneath the nuclei of keratinocytes within the stratum spinosum, where widening of the intercellular spaces is also evident [47].

Other keratin mutations leading to the reaction pattern of *epidermolytic hyperkeratosis*, as found in keratinopathic ichthyosis (former term: bullous congenital ichthyosiform erythroderma of Brocq), epidermolytic palmoplantar keratoderma, epidermolytic acanthoma and others, are also associated with clumping of the keratin skeleton; this only results in tiny eosinophilic intracytoplasmic granules and the vacuolization of the cytoplasm. Although some dehiscence of keratinocytes may be found, cell borders are poorly defined, and intraepidermal blister formation may occur. Additional hallmark features in epidermolytic hyperkeratosis are extensive orthokeratosis, papillomatosis, and acanthosis, along with a prominent granular cell layer containing coarse and irregular keratohyaline granules.

Diseases characterized by the histologic reaction pattern of *acantholytic dyskeratosis*, such as Darier disease, differ by diffusely hypereosinophilic keratinocytes with a pyknotic nucleus and dyskeratotic corneocytes in the horny layer. Likewise, Hailey–Hailey disease shows incomplete acantholysis with polygonal keratinocytes and perinuclear hypereosinophilia but is characterized by a distinctive paleness of the peripheral cytoplasm [48].

#### 5.2.4. Category 4: Detachment of the Stratum Corneum and Flat Acantholytic Corneocytes

In contrast to the previously mentioned forms of desmosomal-type acantholysis, peeling skin syndrome exhibits entirely different histological characteristics. It is marked by the detachment of the stratum corneum and the presence of flat acantholytic corneocytes. Moreover, in the inflammatory variant of generalized peeling skin syndrome (type B) caused by loss of corneodesmosin, the epidermis is hyperplastic with pronounced rete ridges, mild hyperkeratosis, and focal parakeratosis, accompanied by a superficial and perivascular lymphocytic infiltrate. Additionally, a distinct psoriasiform dermatitis may develop, characterized by an elongated papillary dermis with dilated vessels and a thin, compact stratum corneum, without any signs of acantholysis. Ultrastructurally, a loss of corneodesmosomes can be demonstrated, which is associated with *intercellular* splitting of the corneocytes from the stratum granulosum [37].

Immunostaining for corneodesmosin and LEKTI may help to distinguish between PSS type B and other psoriasiform dermatitides, such as Netherton syndrome (Table 4) [37].

Generalized PSS of the non-inflammatory type, designated type A (familial continual skin peeling, keratolysis exfoliativa congenitale), is caused by a mutation in CHST8 [36]. Histologically, a plane of separation either within the lower part of an otherwise normal horny layer or above the granular cell layer is present. Ultrastructural analysis reveals an *intracellular* splitting within the corneocytes [49].

Subcorneal skin splitting can also be found in patients with a homozygous mutation in the Tuftelin-1 (Tuft-1) gene. Tuft-1 plays a role in desmosomal integrity and function, resulting in the perinuclear retraction of intermediate filaments and reduced mechanical stress resistance. Clinically, the patients present with mild peeling skin phenotype, woolly hair, and mild palmoplantar keratoderma but without cardiac involvement [50]. Histological examination reveals a mild acanthosis and hyperkeratosis, widened intercellular spaces and mild acantholysis. Immunofluorescence microscopy demonstrates significantly reduced Tuft-1 staining in the epidermis [51].

From a differential diagnostic perspective, the detachment of the stratum corneum and the presence of flat acantholytic corneocytes in this category of desmosomal-type acantholysis must be distinguished from superficial acantholysis seen in superficial pemphigus, impetigo, and staphylococcal scalded skin syndrome. In these conditions, acantholysis occurs within the stratum granulosum, leading to subcorneal blister formation, sometimes accompanied by an influx of neutrophilic granulocytes.

### 5.3. Solitary Acanthoma and Incidental Findings with Desmosomal-Type Acantholysis

Not surprisingly, we identified the distinct epidermal reaction pattern of desmosomal-type acantholysis both in solitary acanthomas and as an incidental finding in a melanocytic nevus. This alteration resembles other histologic reaction patterns, such as epidermolytic hyperkeratosis, focal acantholytic dyskeratosis, and others, which are observed in genodermatoses, different inflammatory conditions, epidermal nevi, benign acanthomas, dermatofibromas, melanocytic tumours, as well as squamous and basal cell carcinomas (Table 5) [14,15,52,53,54,55]. Likewise, the development of paranuclear eosinophilic globules with widened intercellular spaces in suprabasal keratinocytes in hyaline inclusion acanthoma may possibly be an incidental phenomenon [47].

The occurrence of these histologic reaction patterns is incidental and has no clinical relevance. Despite its distinct microscopic presentation, the underlying cause of this clinically irrelevant yet histologically intriguing phenomenon remains unknown. However, some of these patterns are presumably related to field cancerization and may represent a potentially premalignant change in the peritumoral environment [56].

The histologic patterns of epidermolytic hyperkeratosis and focal acantholytic dyskeratosis have also been reported in epidermal nevi associated with mosaicism, arising from somatic mutations in the keratin genes in the former and the ATP2A2 gene in the latter [1]. While desmosomal-type acantholysis has not yet been detected in epidermal nevi, its occurrence remains feasible.

## 6. Conclusions

Given the significant and, in some cases, life-threatening complications associated with inherited cornification disorders and epidermolysis bullosa, a prompt dermatohistological diagnosis is imperative. Consequently, the establishment of well-defined histological criteria based on validated patterns and algorithms is essential. The knowledge of dermatopathologic changes observed in patients with mutations in desmosomal proteins in routine histology and their differential diagnosis is an important step (Table 6). However, identifying the new pattern of desmosomal-type acantholysis in paraffin sections is challenging due to its limitations and the low sensitivity of routine histology. Instead, the use of semithin sections or electron microscopy is recommended for more precise detection. Additionally, immunohistochemical staining of desmosomal proteins, keratins, or other differential markers can provide valuable diagnostic insights. Ultimately, targeted molecular testing serves as a crucial tool for confirming the diagnosis.

## Figures and Tables

**Table 1:** Desmosome- and corneodesmosome-related genetic diseases.

Molecule	Disease	OMIM	Inheritance
Desmoglein 1	Striate palmoplantar keratoderma, type 1	148700	AD
	Diffuse non-epidermolytic palmoplantar keratoderma	n.a.	AD
	Focal palmoplantar keratoderma	n.a.	AD
	SAM-Syndrome	615508	AR
Desmoglein 2	ARVC 10	610193	AR
Desmoglein 3	Oral and laryngeal mucosal blistering	619226	AR
Desmoglein 4	Localized recessive Hypotrichosis	607903	AR
	Recessive Monilethrix	607903	AR
Desmocollin 2	ARVC11	610476	AD
	ARVC11 with mild PPK and woolly hair	610476	AD/AR
Desmocollin 3	Hypotrichosis with scalp vesicles	613102	AR
Desmoplakin	Striate palmoplantar keratoderma, type 2	612908	AD
	SAM syndrome	n.a.	AR
	ARVC 8	607450	AD
	Palmoplantar keratoderma with left ventricular cardiomyopathy and woolly hair (Carvajal syndrome)	605676	AR/AD
	DCWHK	605676	AR
	EKC syndrome	615821	AD
	Lethal acantholytic epidermal bullosa	609638	AR
Plakoglobin	ARCV12	611528	AD/AR
	Palmoplantar keratoderma with arrhythmogenic right ventricular cardiomyopathy and woolly hair (Naxos syndrome)	601214	AR/AD
	Lethal congenital epidermolysis bullosa	609638	AR
	Acantholytic ectodermal dysplasia	n.a.	AR
Plakophilin 1	Ectodermal dysplasia–skin fragility syndrome	604536	AR
Plakophilin 2	ARCV 9	609040	AD
Corneodesmosin	Generalized inflammatory skin peeling syndrome (PSS1)	270300	AR
	Hypotrichosis simplex	146520	AD

Abbreviations: **AD**: autosomal dominant, **AR**: autosomal recessive, **ARVC**: arrhythmogenic right ventricular cardiomyopathy, **SAM syndrome**: severe dermatitis–multiple allergies–metabolic wasting syndrome, **EKC**: erythrokeratoderma-cardiomyopathy, **DCWHK**: dilated cardiomyopathy with woolly hair and keratoderma, **n.a.**: not assigned.

**Table 2:** Diseases with acantholysis of the desmosomal-type.

Disorder	Mutation of Genes for Desmosomal Proteins	Symptoms
Striate palmoplantar keratoderma, type 1 (SPPK1)	Desmoglein	Striate and island-like palmoplantar keratoderma, exacerbation by mechanical stress, children of two heterozygous mutation carriers are at risk of developing severe dermatitis–multiple allergies–metabolic wasting (SAM) syndrome
Striate palmoplantar keratoderma, type 2 (SPPK2)	Desmoplakin	Striate and island-like palmoplantar keratoderma, exacerbation by mechanical stress
Diffuse palmoplantar keratoderma	Desmoglein	Diffuse palmoplantar keratoderma
Palmoplantar keratoderma with left ventricular cardiomyopathy and woolly hair (Carvajal syndrome)	Desmoplakin	Woolly hair or hypotrichosis, mild striate or diffuse palmoplantar hyperkeratosis, mild ichthyosis, dilated cardiomyopathy
Lethal acantholytic epidermolysis bullosa	Desmoplakin	Lethal generalized epidermolysis, universal alopecia, anonychia, with or without cardiac involvement
Palmoplantar keratoderma with arrhythmogenic right ventricular cardiomyopathy and woolly hair (Naxos syndrome)	Plakoglobin	Woolly hair or hypotrichosis, diffuse or striate palmoplantar hyperkeratosis, arrhythmogenic right ventricular cardiomyopathy (ARVC)
Acantholytic ectodermal dysplasia	Plakoglobin	Epidermal skin fragility, diffuse palmoplantar keratoderma, curly hair, nail dystrophy, no cardiac symptoms at younger age
Lethal congenital epidermolysis bullosa	Plakoglobin	Generalized epidermolysis, massive transcutaneous fluid loss, no cardiac dysfunction
Ectodermal dysplasia/skin fragility syndrome	Plakophilin	At-birth peeling and blistering skin on soles, progression to painful palmoplantar hyperkeratosis, anomalies of hair, nail dystrophy, failure to thrive
Severe dermatitis–multiple allergies–metabolic wasting (SAM) syndrome	Desmoglein or desmoplakin	Ichthyosiform erythroderma, failure to thrive, hypotrichosis, nail dystrophy, eosinophilia, elevated IgE levels, recurrent infections
Erythrokeratodermia-cardiomyopathy syndrome	Desmoplakin	Erythrokeratodermia, palmoplantar keratoderma, scant woolly hair, tooth defects, onychodystrophia, cardiomyopathy

**Table 3:** Histologic spectrum of desmosomal-*type* acantholysis (four categories).

Desmosomal-Type Acantholysis	Diseases Related to Mutations of Genes for Desmosomal Proteins	Differential Diagnoses
**Category 1** Widening of the intercellular spaces in the stratum spinosum and occasionally the stratum granulosum	SPPK, types 1 and 2, diffuse NEPPK, Palmoplantar keratoderma with left ventricular cardiomyopathy and woolly hair (Carvajal syndrome), Lethal acantholytic epidermolysis bullosa, Palmoplantar keratoderma with arrhythmogenic right ventricular cardiomyopathy and woolly hair (Naxos syndrome), Acantholytic ectodermal dysplasia, Lethal congenital epidermolysis bullosa, Incidental finding in acanthoma and melanocytic naevi	Pemphigus diseases Herpes viruses and other viral infections, Eczematous diseases and other spongiotic dermatitides Artefacts
**Category 2** Widening of the intercellular spaces with psoriasiform dermatitis	SAM syndrome, Erythrokeratodermia-cardiomyopathy syndrome	Netherton syndrome CHILD syndrome MALT1 deficiency syndrome
**Category 3** Widening of intercellular spaces with an intracytoplasmic hypereosinophilic globule in suprabasal keratinocytes	Ectodermal dysplasia–skin fragility syndrome, Erythrokeratodermia-cardiomyopathy syndrome	Pachyonchia congenita Keratinopathic Ichthyosis Epidermolytic palmoplantar keratoderma Epidermolytic acanthoma Darier disease Hailey–Hailey disease
**Category 4** Detachment of the stratum corneum and flat acantholytic corneocytes	Peeling skin syndrome, type A and B	Skin fragility, woolly hair, and palmoplantar keratoderma related to mutation in the TUFT1 gene Pemphigus foliaceus Impetigo Staphylococcal scaled skin disease

Abbreviations: **SAM syndrome**: Severe dermatitis–multiple allergies–metabolic wasting syndrome, **SPPK**: Striate palmoplantar keratodermas, **NEPPK**: non-epidermolytic palmoplantar keratoderma, **TUFT1**: Tuftelin-1 (enamel-associated protein).

**Table 4:** Ichthyoses with inflammatory psoriasiform pattern.

Disease	Key Protein
SAM syndrome	Desmoglein, Desmoplakin
Erythrokeratodermia-cardiomyopathy syndrome	Desmoplakin
Ectodermal dysplasia–skin fragility syndrome	NSDHL (Plakophilin *)
Generalized inflammatory skin peeling syndrome	Corneodesmosin
Netherton syndrome	LEKTI
CHILD syndrome	Adipophilin
MALT 1 deficiency syndrome	MALT 1

* Plakophilin serves as an immunohistochemical marker on paraffin sections, aiding in the visualization of the characteristic xanthomatized macrophages in the papillary dermis.

**Table 5:** Epidermal reaction patterns found in genodermatoses, epidermal nevi, acquired skin diseases or as an incidental finding.

Focal acantholytic dyskeratosis Focal acantholysis Epidermolytic hyperkeratosis Clear cell acanthosis Cornoid lamella Follicular mucinosis Hailey–Hailey-like pattern of acantholysis (new, personal observation) Desmosomal-type acantholysis (new)

**Table 6:** Histologic differential diagnoses of genodermatosis with desmosomal-type acantholysis.

Diagnoses	Key Histologic Features
Pemphigus vulgaris ^1^	Roundish keratinocytes with pyknotic nuclei (complete acantholysis); intraepidermal blistering with intact supraepidermal roof and normal cornification
Pemphigus foliaceus/Impetigo/Staphylococcal scalded skin disease ^2^	Detachment of the stratum corneum; flat acantholytic keratinocytes in the stratum granulosum
Keratinopathic ichthyosis/Epidermolytic acanthoma/Epidermolytic palmoplantar keratoderma ^3^	Acanthotic epidermis; vacuolated and cytolytic keratinocytes with hypereosinophilic granules; irregular keratohyalin granules; orthohyperkeratosis
Darier disease	Focal suprabasal acantholysis; keratinocytes with pyknotic nuclei and hypereosinophilic cytoplasm (dyskeratosis); hyperkeratosis with parakeratosis
Hailey–Hailey disease	Widening of intercellular spaces; polygonal keratinocytes with regular nuclei (incomplete acantholysis) and perinuclear hypereosinophilia with pale cell periphery
Herpes infection	Roundish keratinocytes (fully acantholytic cells); ballooning degeneration (intracellular edema); steel-grey nuclei with marginal chromatin; multinucleated keratinocytes
Eczema (spongiotic dermatitis)	Widening of intercellular spaces; pale keratinocytes with intact nuclei; vesicle/blister formation; serous exudation; parakeratosis and crusting in late stages

**Footnotes: Differential diagnosis with regard to the four categories of desmosomal-type acantholysis**. ^1^ Desmosomal-type acantholysis, category 1 and 2: Widening of intercellular spaces—polygonal keratinocytes with regular nuclei (incomplete acantholysis) and (hyper-)eosinophilic cytoplasm; additional psoriasiform dermatitis in group 2. ^2^ Desmosomal-type acantholysis, category 4: Detachment of the stratum corneum—flat acantholytic corneocytes. ^3^ Desmosomal-type acantholysis, category 3: Widening of intercellular spaces with intracytoplasmic hypereosinophilic globules in suprabasal keratinocytes.

## Data Availability

All data from this study is provided within the manuscript.
